# Endoscopy-assisted surgery for the management of benign breast tumors: technique, learning curve, and patient-reported outcome from preliminary 323 procedures

**DOI:** 10.1186/s12957-016-1080-5

**Published:** 2017-01-11

**Authors:** Hung-Wen Lai, Hui-Yu Lin, Shu-Ling Chen, Shou-Tung Chen, Dar-Ren Chen, Shou-Jen Kuo

**Affiliations:** 1 Endoscopic and Oncoplastic Breast Surgery Center, Changhua Christian Hospital, 135 Nanxiao Street, Changhua, 500 Taiwan; 2 Division of General Surgery, Changhua Christian Hospital, Changhua, Taiwan; 3 Department of Surgery, Comprehensive Breast Cancer Center, Changhua Christian Hospital, Changhua, Taiwan; 4 School of Medicine, National Yang Ming University, Taipei, Taiwan; 5 Division of Breast surgery and General Surgery, Department of Surgery, Cardinal Tien Hospital, Xindian Dist., New Taipei City, Taiwan

**Keywords:** Endoscopy-assisted breast surgery (EABS), Minimally invasive breast surgery, Benign breast lesions

## Abstract

**Background:**

Endoscopy-assisted breast surgery (EABS), a technique that optimizes cosmetic outcome because it is performed through small wounds hidden in inconspicuous areas, could be an alternative surgical technique for benign breast tumors. In this study, we report the preliminary results of 323 EABS procedures performed at our institution for the management of benign breast tumors.

**Methods:**

The medical records of patients who underwent EABS for benign breast lesions during the periods August 2010 to December 2015 were collected from the Changhua Christian Hospital EABS database. Data on clinicopathologic characteristics, type of surgery, hospital stay, and complications were analyzed to determine the effectiveness of the procedure for benign breast tumors. The operating time with the number of procedure performed was analyzed for learning curve evaluation. Patient satisfaction with cosmetic outcome was evaluated with a self-report questionnaire.

**Results:**

A total of 323 EABS procedures were performed in 286 patients with benign breast lesions, including 249 (90.5%) patients with unilateral lesions. The mean age was 36 years, the mean tumor size was 2.2 cm, and the mean distance from the nipple to the tumor was 5.2 cm. Most (93.8%, 303/323) of these tumors were excised through a transareolar wound, 2.4% (8/323) through an axillary wound, and 0.3% (1/323) through the infra-mammary fold. Histopathologic analysis revealed that 63.5% (202/318) of the tumors were fibroadenoma-related lesions. The mean operative time was 81.4 min (59~89 min), which was decreased with experience increased. The overall rate of complications was 6.5%, and all were minor and wound-related. Among the 110 patients who participated in the self-report cosmetic outcome evaluation, 85.4% reported being satisfied with the cosmetic result, and almost all were satisfied with breast symmetry. Of the patients interviewed, 92.7% reported that they would choose the same procedure if they had to undergo the operation again.

**Conclusions:**

Our preliminary results show that transareolar video-assisted breast surgery is a safe and effective procedure with good cosmetic outcome and that it could be appropriate for patients with moderate to large peripherally located breast tumors.

**Trial registration:**

CCH-IRB No.15115. Registered 14 December 2015 (retrospectively registered).

## Background

Breast masses in young patients are usually benign in nature and are most commonly fibroadenomas, cysts, or fibrocystic lesions [1, 2]. Management of benign breast tumors ranges from non-operative conservative treatment to surgical excision [3–7]. Asymptomatic tumors are often managed with observation [3, 5, 7]; however, patients who present with a progressively enlarging mass, a tumor that is atypical in presentation, or with breast deformity may request surgical excision.

The goal of surgical excision of benign breast tumors is the complete excision of the tumor with a thin rim of normal tissue [1, 5, 7]. Most (70%) breast tumors are single and unilateral, which allows for volume-dependent local excision to be carried out safely [1, 5, 7]. Usually, the normal parenchyma will reestablish its shape and achieve subsequent symmetry with time [8]. Although conventional excision is a safe and effective method for managing benign breast tumors, the procedure can result in suboptimal cosmetic outcomes such as conspicuous scars and misshapen breasts caused by cavity collapse after resection of the tumor.

Endoscopy-assisted breast surgery (EABS), a technique performed through minimal axillary and/or periareolar incisions, was initially developed to facilitate breast augmentation [9–11] but is now increasingly used to excise benign breast tumors [12–14], resect malignant breast tumors [15–18], and assist in sentinel lymph node biopsy [19, 20]. EABS, a modality that optimizes cosmetic outcome because it is performed through small wounds hidden in inconspicuous areas, could be an alternative surgical technique for the management of benign breast tumors.

In this study, on our preliminary experience performing 323 EABS procedures for the management of benign breast tumors, we present the technique of video-assisted breast surgery, the indications for surgery, the preliminary outcomes, the learning curve associated with EABS, and the patient-reported cosmetic outcomes.

## Methods

Data on patients with benign breast diseases who underwent endoscopy-assisted surgery at the Changhua Christian Hospital during the periods August 2010 to December 2015 were collected from the hospital’s EABS database. The data included clinicopathologic characteristics, type of surgery, operation time, blood loss, hospital stay, and complications. The data collection was performed by a specially trained nurse (SLC), and the correctness of the data was checked by the principal investigator (HWL). This study was approved by the institutional review board (IRB) of the Changhua Christian Hospital (IRB No. 151115).

### Patient selection criteria

**Fig. 1:**
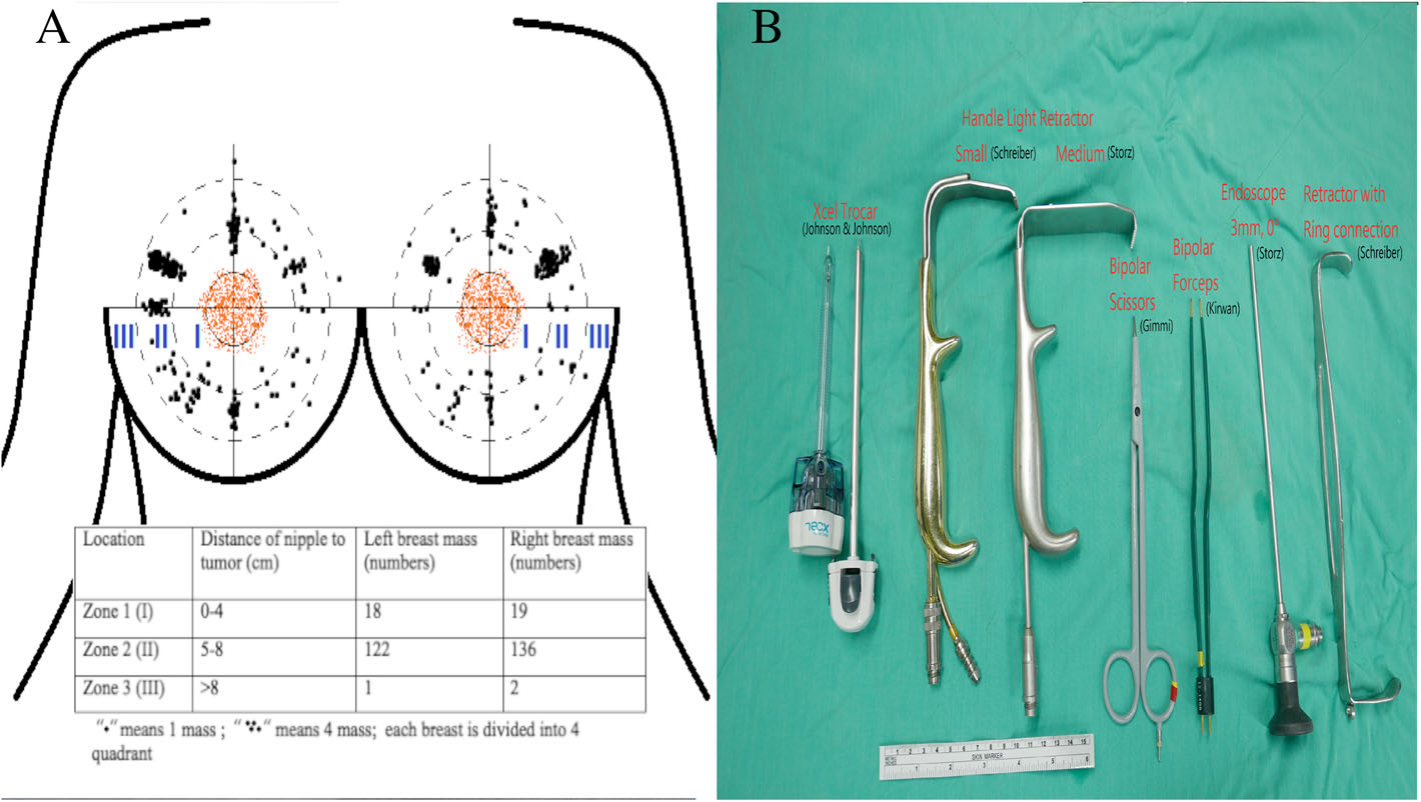
**a** Illustration and classification of breast tumor location according to different anatomic zones. **b** Instruments

**Fig. 2:**
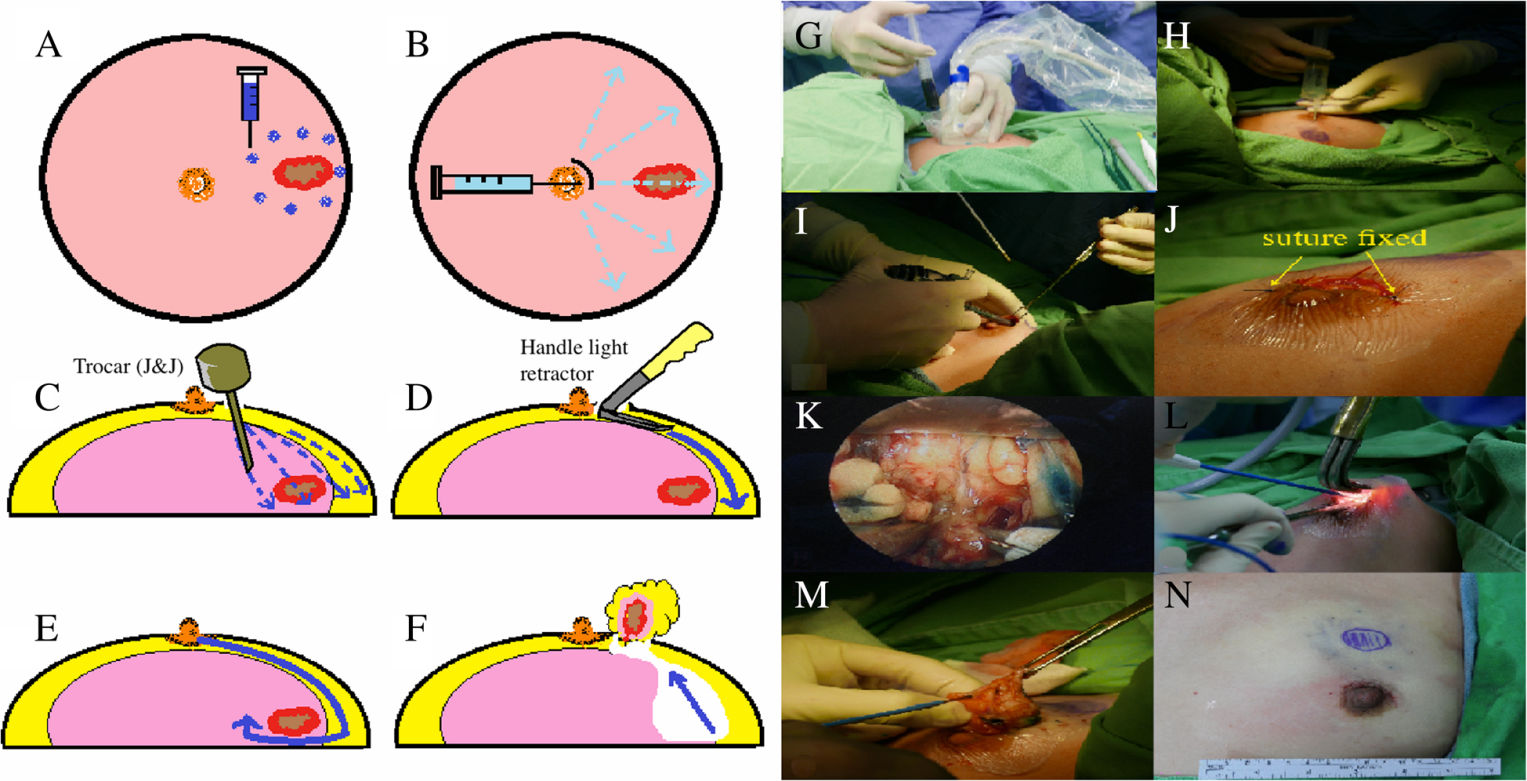
Illustration of surgical procedures: **a** Mark the planned resection line on the mammary gland by gel containing blue dye. **b** Hydrodissection of the subcutaneous layer with tumescent solution. **c** Tunneling method. **d** Subcutaneous flap creation (under endoscopic guidance). **e** Posterior wall fascia dissection (with handle light retractor). **f** Removal of the specimen. **g** Marking the planned resection by gel containing blue dye. **h** Tumescent solution injection. **i** Tunnel creation. **j** Hand-made wound edge protector (waterproof sticker fixed by running suture with 4–0 nylon). **k** Flap creation (under endoscopic guidance). **l** Posterior wall fascia dissection (with handle light retractor). **m** Removal of the specimen. **n** Postoperative gross picture

Preoperative sonograms and/or mammograms were used to determine the eligibility of patients for EABS. Indications for EABS included a tumor size greater than 2 cm located in zone II or III (Fig. 1a) and or tumor size 1–2 cm with patient’s preferences. Zone I breast tumors were excised with or without endoscopic guidance. Patients for whom EABS was contraindicated included patients with severe comorbid conditions, such as heart disease, renal failure, liver dysfunction, and poor performance status as assessed by the primary physicians. The instruments used during endoscopic benign breast excision are illustrated in Fig. 1b and summarized in the endoscopy-assisted breast surgery technique section (Fig. 2).

### Learning curve evaluation

To evaluate the learning curve associated with EABS and survey whether the operating time would decrease with experience, we compared the operating time in different periods. Initially, we listed each procedure with corresponding operating time, which includes both of the unilateral and bilateral breast surgeries. Then, we divided all of the unilateral procedures into five groups of 43–50 procedures per group (only single-sided surgery was included to prevent bias from bilateral surgeries). Finally, we analyzed the first 50 and last 50 EABS procedures to see the difference in operating time between the initial phase and the mature phase.

### Esthetic outcome evaluation

The cosmetic outcome was evaluated objectively by patients with a self-report questionnaire 2 months after the operation when their wounds had healed. The patients were asked to compare the pre and postoperative breast shape, nipple position, and volume symmetry of both breasts.

The self-report questionnaire comprised three questions:

1. Are you satisfied with the cosmetic outcome? The esthetic result was graded as “very good,” “good,” “fair,” and “poor.” Patients who reported “very good” or “good” results were defined as being satisfied with the outcome.
2. How do you feel about the symmetry of the breast after surgery? The result was graded as “symmetric,” “mildly asymmetric,” “moderately asymmetric,” and “poor.”
3. Would you choose the same operation if you required the surgery again? The possible responses were “yes,” “not sure,” and “no.”

### Endoscopy-assisted breast surgery technique

Details of the surgical technique for EABS used at our hospital have been described previously [21, 22]. Briefly, after preoperative marking, the patient was placed in a supine position, and the arm was adducted for the areolar approach or was abducted 90° for the axillary approach to avoid disturbing the operative procedure. Intraoperative sonography was routinely used to identify the location of breast tumors, and a small amount of gel containing blue dye was injected to mark the planned resection line on the mammary gland to ensure the adequate removal of breast tumors, which sometimes were non-palpable (Fig. 2a, g).

Endoscopic video monitors (Olympus Optical Co., Tokyo, Japan) were set up on both sides of the patient’s head and watched by two surgeons. An ended, ridged endoscope measuring 3 mm in diameter with a viewing angle of 0° was used in all procedures. A tumescent solution (lactated Ringer’s solution 100 ml containing 5 ml sodium bicarbonate, 20 ml 1% lidocaine, and epinephrine 0.5 ml (1:1000)) was injected subcutaneously into the whole breast to minimize bleeding (Fig. 2b, h).

A periareolar skin incision (one third or semi-periareolar) or axillary incision (when the tumor was located near the axilla) was made. A skin flap measuring 3–5 mm in thickness was created using an Xcel optical bladeless trocar (Johnson & Johnson, Tokyo, Japan) under endoscopic guidance or an Xcel trocar blindly (Fig. 2c, i). The wound edge protector, which is a waterproof patch fixed with 4–0 nylon sutures, was placed to prevent wound maceration (Fig. 2j).

The septa between the skin flap and parenchyma were dissected under direct vision initially and then using bipolar scissors (Powers Star, Johnson & Johnson KK) under endoscopic guidance. The subcutaneous flap was dissected beyond the blue hue tissue under endoscopic guidance to create the work space (Fig. 2k). After enough space had been created, the breast parenchyma was resected with an electrocoagulater and a light retractor under direct vision (Fig. 2d, l). Tumor specimens were removed through the periareolar wound. Once all tumors had been resected, the gland was reconstructed by undermining, advancing, and performing a layered closure of the flanking glandular breast tissue using 3–0 absorbable sutures [2]. The wound protector was removed, and the wound was closed with 3–0 polysorb and 4–0 monocryl sutures (Fig. 2n).

### Statistical analyses

Data are reported as means ± standard deviation (SD). Differences in continuous variables were tested by the independent *t* test. The chi-square test was used for categorical comparisons of data when appropriate. A *p* value of less than 0.05 was considered to indicate statistical significance; all tests were two-tailed. All statistical analyses were performed with the statistical package SPSS (Version 19.0, SPSS, Chicago).

## Results

From August 2010 to December 2015, a total of 323 EABS procedures were performed in 286 patients; the vast majority of whom had unilateral disease (*n* = 249, 90.5%). The mean age of the patients was 36 ± 13 years (range, 13–69 years), and the mean tumor size was 2.2 ± 1.1 cm (range, 0.9 to 6.8 cm). Most of the tumors (93.8%, 303/323) were excised through a transareolar wound, 2.4% (8/323) through an axillary wound, and 0.3% (1/323) through the infra-mammary fold. The mean distance from the nipple to the tumor was 5.2 cm (2–9 cm) (Fig. 1a). The average number of tumors excised was 1.0 ± 0.2 (range 1–3) per breast. The clinical manifestations of patients with benign breast tumors who received EABS are summarized in Table 1.

**Table 1:** Clinical manifestations of patients received endoscopy-assisted breast surgery for benign disease

	*N* = 286 patients, 323 EABS
Patient	
Gender (female)	323 (100%)
Age (years old)	36 ± 13 (range 13–69)
<20	31 ± 1(9.5%)
20–40	175 ± 6 (54.1%)
>40	117 ± 6 (36.2%)
Location	
Unilateral	249 (90.5%)
Right	169 (52.3%)
Left	154 (47.6%)
Bilateral	37 (9.4%)
Distance to nipple (cm)	5.22 ± 1.14 cm (2–9 cm)
Characteristics of mass	
Sonogram mean tumor size	2.9 ± 3.93 cm (0.62–20 cm)
<2 cm	150 (46%)
>2 cm	136 (42%)
Pathology mean tumor size	2.2 ± 1.05 cm (0.9–6.8 cm)
Specimen weight (g)	30.1 ± 27.5 g (7–133 g)
Number of mass per breast	1.04 ± 0.22 (range 1–3)
0 (microcalcification)	14
1	180
1 + microcalcification	2
2	4
3	1
N/A	78

*N/A* not applicable

The mean operating time was 81.4 ± 30.0 min, the mean blood loss was 12.0 ± 9.1 ml, and the mean hospital stay was 2.2 ± 0.4 days (Table 2). Most patients had a smooth postoperative recovery, and there were no major perioperative complications. Overall, the rate of complications associated with EABS was 6.5% (21/323), and all were minor and wound-related. Two (0.6%) procedures resulted in wound infection, which resolved after antibiotic therapy, four (1.2%) procedures resulted in hematoma, and four (1.2%) procedures resulted in poor wound healing during outpatient follow-up (Table 2).

**Table 2:** EABS procedures detail

Incision location	
Areolar	303 (93.8%)
Axillary	8 (2.4%)
Infra-mammary fold	1 (0.3%)
Operation time	
Mean operation time (min)	81.4 ± 30.0 min
Single	90 ± 20.6 min (35–150 min)
Bilateral	121.8 ± 44.53 min (70–340 min)
Blood loss evaluation	
Blood loss (ml)	12.0 ± 9.1 ml
Single	10.57 ± 3.12 ml (3–30 ml)
Bilateral	21.05 ± 21.37 ml (5–140 ml)
Complication	21 (6.5%)
Hematoma	4 (1.2%)
Wound infection	2 (0.6%)
Seroma	11 (3.4%)
Poor wound healing	4 (1.2%)

Of the 323 EABS procedures performed, 163 (50%) were performed in patients who received biopsy before the operation. Of those procedures, 144 (44.5%) were performed in patients who received core needle biopsy (CNB), 18 (5.5%) were performed in patients who received fine needle aspiration cytology (FNAC), and one was performed in a patient who received both CNB and FNAC (0.3%). The postoperative pathologic reports revealed 156 (49%) fibroadenomas, 46 (16.6%) fibroadenoma specimens with fibrocystic change, 55 (17%) specimens with fibrocystic change, and five (2%) malignant specimens. Of the 5 patients with malignant disease, CNB showed evidence of benign breast lesion in 2 patients, and microcalcification was detected in 3 patients before the operation. All 5 patients showed negative margins after the initial EABS procedure, and 1 patient had a margin distance <1 mm. The 4 patients with margins >1 mm did not receive further re-excision, and the patient with a margin <1 mm received further wide excision. The pathology report showed no residual breast cancer. The pathology reports associated with the 323 EABS procedures are summarized in Table 3.

**Table 3:** Pathology characteristics

*N* = 323	
Benign	318 (98%)
Fibroadenoma	143 (44.2%)
Cellular fibroadenoma	4 (1.2%)
Complex fibroadenoma	3 (0.9%)
Juvenile fibroadenoma	6 (1.8%)
Fibroadenoma + fibrocystic change	46 (16.6%)
Fibrocystic change	55 (17%)
Fibrocystic change + adenosis	7 (2.2%)
Phyllodes tumor	10 (3.1%)
Intraductal papilloma	11 (3.4%)
Harmatoma	6 (1.8%)
Adenosis	3 (0.9%)
Others	24 (7.5%)
Malignancy	5 (2%)
DCIS in a fibroadenoma	1 (0.3%)
IDC	1 (0.3%)
LCIS	2 (0.6%)
LCIS + DCIS	1 (0.3%)
Positive margin of malignancy	0/5 (0%)

**Fig. 3:**
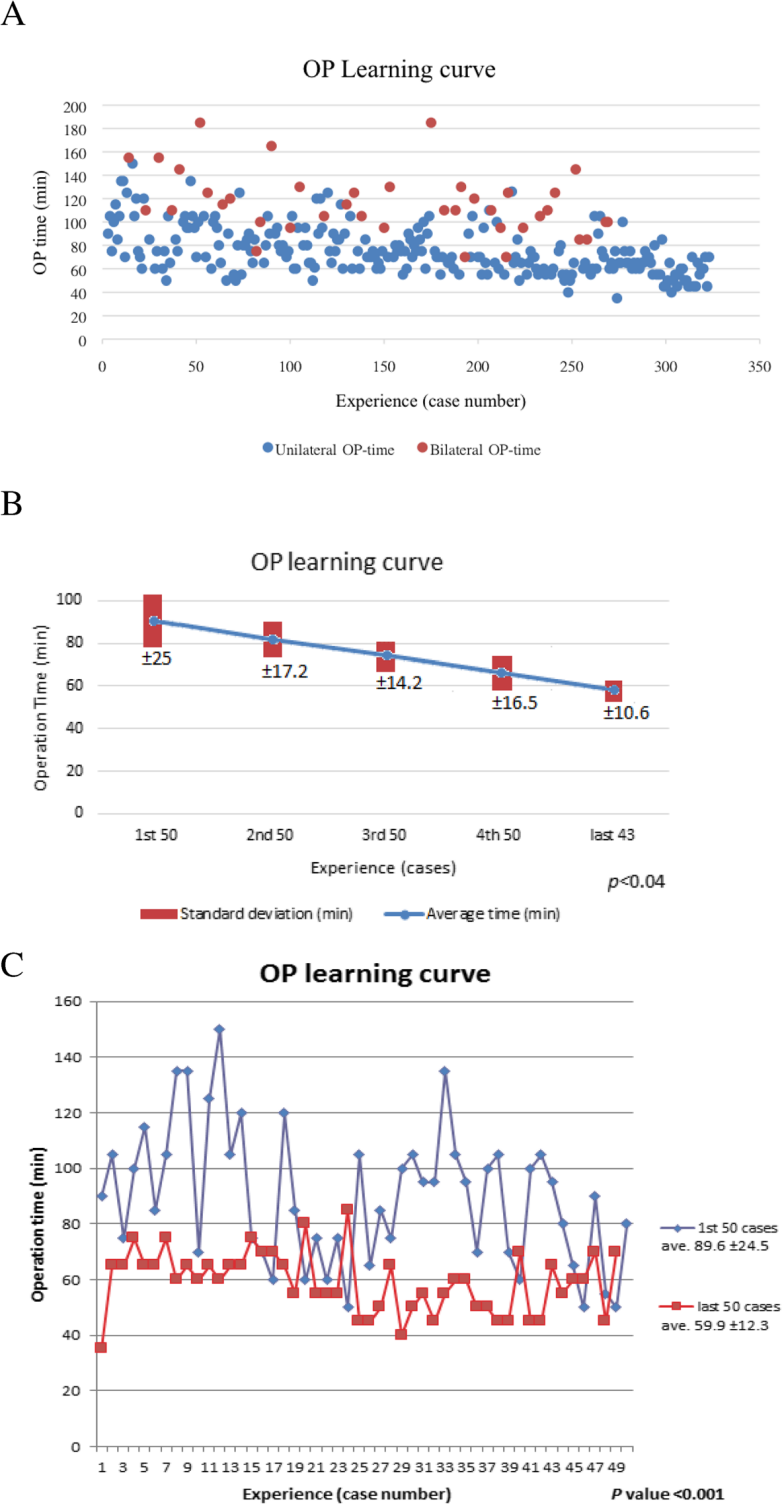
Operation learning curve. **a** Unilateral vs. bilateral OP learning curve. **b** Unilateral OP time: divided into five groups (43–50 procedures in each group). Comparison of the average OP time and standard deviation in each group (*p* < 0.04). **c** First 50 procedures vs. last 50 procedures in the OP learning curve

To evaluate the learning curve associated with EABS and survey whether the operating time would decrease with experience, we compared the operating time with the number of procedures performed. We observed a trend in decreasing operating time with experience (Fig. 3). There was also a trend in decreasing operating time when we grouped the operations into five groups according to the sequence in which they were performed (*p* < 0.04, Fig. 3b). There was a significant difference in operating time between the first 50 procedures (the initial learning phase, mean operating time 89.6 ± 24.5 min) and the last 50 procedures (the mature phase, mean operating time 59.9 ± 12.3 min, *p* < 0.001, Fig. 3c).

**Fig. 4:**
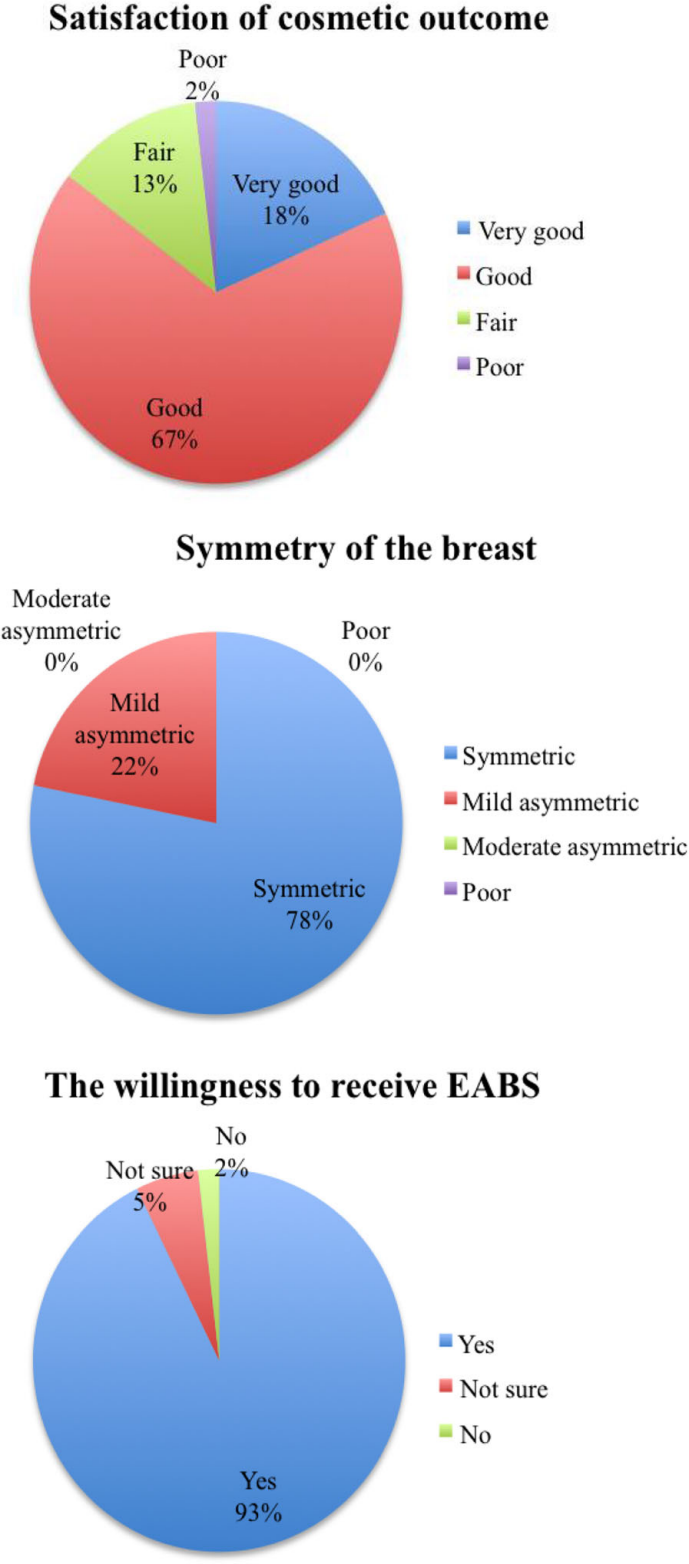
Postoperative cosmetic outcome evaluation

A total of 110 (38.5%) patients completed the postoperative cosmetic outcome questionnaire. A “very good” cosmetic outcome was reported by 20 (18.4%) patients, a “good” outcome was reported by 74 (67.2%) patients, a “fair” outcome was reported by 14 (12.7%) patients, and a poor outcome was reported by 2 (1.8%) patients (Fig. 4 a). Of the 110 patients, 86 (78.1%) reported that the surgery resulted in a symmetrical breast, and 24 (21.8%) reported that their breast was mildly asymmetric after the surgery (Fig. 4b). Among the 24 patients who reported mild asymmetry, 9 (37.5%) reported dimpling of the breast contour, 7 (29.1%) complained that breast size became smaller after the operation, and 1 (4.1%) patient felt that the contralateral breast (healthy breast) was mildly ptotic compared with the operated breast. In regard to the willingness to perform the same procedure if they had to undergo a second operation, the majority (*n* = 102, 92.7%) reported that they would choose the same procedure, 6 (5.5%) patients were not sure, and 2 (1.8%) patients reported that they would not want to receive the EABS procedure either because of wound pain or because it resulted in fair cosmetic outcome (Fig. 4c).

## Discussion

In this study, we reported our preliminary experience with performing endoscopy-assisted breast surgery for managing of benign breast tumors. In our analysis of the 323 EABS procedures performed in 286 patients with benign breast lesions, we found that most of the patients were young (mean age 36 years, 63.8% <40 years) and had moderate to large tumors (mean 2.2 cm) located far away from the areolar complex (mean 5.2 cm, Fig. 1a).

Fibroadenoma is the most common histologic subtype of benign breast tumors. In our patients, 63.5% (202/318) of the lesions were fibroadenomas. Newer techniques have been proposed for smaller fibroadenomas, such as percutaneous excision [6, 23] or in situ cryoablation [4, 24], which are less invasive than conventional excision. Percutaneous excision performed with the Mammotome or Encor system has been shown to be appropriate for excising small or non-palpable masses [6, 23]. Cryoablation, which achieves complete ablation of the breast mass by the “freeze-thaw-freeze” technique without the need for subsequent resection, has been approved by the FDA for the treatment of fibroadenoma [4, 24]. However, surgical excision remains the standard treatment for large fibroadenomas [5, 7], giant fibroadenomas [25, 26], juvenile fibroadenomas [27], phyllodes tumors [28], and multiple fibroadenomas [29, 30].

A circumareolar incision, which leaves the least visible scar, is a common approach for the management of benign breast lesions [1, 29, 31, 32]. The cosmetic result is favorable, and the tumor can usually be adequately resected. Even giant fibroadenomas have been shown to be successfully removed through a circumareolar incision using the “Swiss-Roll” operation [31, 32]. However, not all fibroadenomas, such as those located far away from the areolar complex, can be managed through a simple circumareolar incision. An incision above the lesion can result in suboptimal esthetic outcomes and is one of the important reasons why patients are often reluctant to receive surgical excision for breast tumors even when the disease is symptomatic.

EABS for breast tumors was first used in 1998, when Kitamura et al. [33] first reported the use of endoscopic surgery with three small incisions in the mid-axillary line for removal of benign breast tumors. A modified retromammary space approach was proposed by Osanai et al. [12, 34] in 2001 and was modified by Liu et al. [14] in 2009 with two or three small incisions located in the axilla and/or areolar complex. In the current study, a video-assisted transareolar approach was used to excise most (97%) of the benign breast tumors. This technique was initially proposed by Tamaki et al. [15] in 2001 as a minimally invasive surgical technique that involves performing the surgery through a single periareolar incision. This technique was refined by Cheng et al. [35] to excise giant juvenile fibroadenomas (5–10 cm in size) with subsequent retrieval of the specimen with an endoscopic plastic bag through the periareolar incision.

Transareolar video-assisted breast surgery (as illustrated in Fig. 2) can be used to excise moderate to large breast tumors (>2 cm) that are located far from the areolar complex (>4 cm). One of the benefits of the procedure is that it only leaves a small inconspicuous scar over the areola and better cosmetic outcome for patient with multiple tumors. In this study, 85.4% (94/110) of patients reported being satisfied with the cosmetic result, and all patients reported that they were satisfied with breast symmetry after the procedure (Fig. 4). A very high (92.7%) proportion of patients reported that they would choose the same procedure if they needed surgery in the future.

The main limitation of performing endoscopy-assisted breast surgery is the need for general anesthesia and hospitalization. In our study, the mean hospital stay was 2.2 ± 0.4 days as patients chose to be discharged the day after operation because of insurance concerns (Table 2). In fact, some patients were discharged the same day after recovery from anesthesia. The relatively long operating time (mean 81.4 ± 30 min) is another limitation for the widespread use of this surgical technique for benign breast tumors. As we showed in the learning curve study (Fig. 3), the operating time decreased significantly from 89.6 ± 24.5 min in August 2010 when we first started to perform the procedure to 59.9 ± 12.3 min in December 2015 (*p* < 0.001). Among the 323 EABS procedures performed, 5 (2%) were performed in patient who found to have malignancy in the final pathologic report (Table 3). All of the specimens had negative margins, and only one patient, who had peripheral margin <1 mm, received further re-excision, and no residual breast cancer was found. This indicates that EABS is an effective and oncologically safe procedure for the management of breast tumors.

Currently, there is no standard for the management of benign breast tumors [36]. Video-assisted surgery via a circumareolar incision is a useful technique for peripherally located tumors. It not only allows for excision of the lesions but also results in an inconspicuous postoperative scar and hence better cosmetic outcome.

## Conclusions

Our preliminary results show that transareolar video-assisted breast surgery is a safe and effective procedure with good cosmetic outcome, especially for patients with moderate to large peripherally located breast tumors.

## Abbreviations

EABS: Endoscopy-assisted breast surgery

## References

[1] Chang DS, McGrath MH (2007). Management of benign tumors of the adolescent breast. Plast Reconstr Surg.

[2] Anderson BO, Masetti R, Silverstein MJ (2005). Oncoplastic approaches to partial mastectomy: an overview of volume-displacement techniques. Lancet Oncol.

[3] Cant PJ, Madden MV, Coleman MG, Dent DM (1995). Non-operative management of breast masses diagnosed as fibroadenoma. Br J Surg.

[4] Edwards MJ, Broadwater R, Tafra L, Jarowenki D, Mabry C, Beitsch P, Whitworth P, Martin RC, Oetting L (2004). Progressive adoption of cryoablative therapy for breast fibroadenoma in community practice. Am J Surg.

[5] Carty NJ, Carter C, Rubin C, Ravichandran D, Royle GT, Taylor I (1995). Management of fibroadenoma of the breast. Ann R Coll Surg Engl.

[6] Grady I, Gorsuch H, Wilburn-Bailey S (2008). Long-term outcome of benign fibroadenomas treated by ultrasound-guided percutaneous excision. Breast J.

[7] Greenberg R, Skornick Y, Kaplan O (1998). Management of breast fibroadenomas. J Gen Intern Med.

[8] Jacob MM (2000). Application of reduction mammaplasty in treatment of giant breast tumour. Br J Plast Surg.

[9] Eaves FF, Bostwick J, Nahai F, Murray DR, Styblo TM, Carlson GW (1995). Endoscopic techniques in aesthetic breast surgery: augmentation, mastectomy, biopsy, capsulotomy, capsulorrhaphy, reduction, mastopexy, and reconstructive techniques. Clin Plast Surg.

[10] Momeni A, Padron NT, Bannasch H, Borges J, Bjorn Stark G (2006). Endoscopic transaxillary subpectoral augmentation mammaplasty: a safe and predictable procedure. J Plast Reconstr Aesthet Surg.

[11] Ulusal BG, Cheng MH, Wei FC (2006). Simultaneous endoscope-assisted contralateral breast augmentation with implants in patients undergoing postmastectomy breast reconstruction with abdominal flaps. Plast Reconstr Surg.

[12] Osanai T, Nihei Z, Ichikawa W, Sugihara K (2002). Endoscopic resection of benign breast tumors: retromammary space approach. Surg Laparosc Endosc Percutan Tech.

[13] Yamashita K, Shimizu K (2006). Endoscopic video-assisted breast surgery: procedures and short-term results. J Nihon Med Sch.

[14] Liu H, Huang CK, Yu PC, Chen HP, Hsieh PM, Hung KC, Hung CM, Chen YS (2009). Retromammary approach for endoscopic resection of benign breast lesions. World J Surg.

[15] Tamaki Y, Sakita I, Miyoshi Y, Sekimoto M, Takiguchi S, Monden M, Noguchi S (2001). Transareolar endoscopy-assisted partial mastectomy: a preliminary report of six cases. Surg Laparosc Endosc Percutan Tech.

[16] Lee EK, Kook SH, Park YL, Bae WG (2006). Endoscopy-assisted breast-conserving surgery for early breast cancer. World J Surg.

[17] Sakamoto N, Fukuma E, Higa K, Ozaki S, Sakamoto M, Abe S, Kurihara T, Tozaki M (2009). Early results of an endoscopic nipple-sparing mastectomy for breast cancer. Ann Surg Oncol.

[18] Nakajima H, Fujiwara I, Mizuta N, Sakaguchi K, Hachimine Y (2009). Video-assisted skin-sparing breast-conserving surgery for breast cancer and immediate reconstruction with autologous tissue. Ann Surg.

[19] Yamashita K, Shimizu K (2009). Evaluation of sentinel lymph node metastasis alone guided by three-dimensional computed tomographic lymphography in video-assisted breast surgery. Surg Endosc.

[20] Yamashita K, Shimizu K (2009). Video-assisted breast surgery can sample the second and third sentinel nodes to omit axillary node dissection for sentinel-node-positive patients. Surg Endosc.

[21] Lai HW, Wu HS, Chuang KL, Chen DR, Chang TW, Kuo SJ, Chen ST, Kuo YL (2015). Endoscopy-assisted total mastectomy followed by immediate pedicled transverse rectus abdominis musculocutaneous (TRAM) flap reconstruction: preliminary results of 48 patients. Surg Innov.

[22] Lai HW, Chen ST, Chen DR, Chen SL, Chang TW, Kuo SJ, Kuo YL, Hung CS (2016). Current trends in and indications for endoscopy-assisted breast surgery for breast cancer: results from a six-year study conducted by the Taiwan endoscopic breast surgery cooperative group. PLoS One.

[23] Sperber F, Blank A, Metser U, Flusser G, Klausner JM, Lev-Chelouche D (2003). Diagnosis and treatment of breast fibroadenomas by ultrasound-guided vacuum-assisted biopsy. Arch Surg.

[24] Fine RE, Staren ED (2006). Percutaneous radiofrequency-assisted excision of fibroadenomas. Am J Surg.

[25] Hoffman SH (1978). Giant fibroadenoma of the breast: immediate reconstruction following excision. Br J Plast Surg.

[26] Simmons RM, Cance WG, Iacicca MV (2000). A giant juvenile fibroadenoma in a 12-year-old girl: a case for breast conservation. Breast J.

[27] Wechselberger G, Schoeller T, Piza-Katzer H (2002). Juvenile fibroadenoma of the breast. Surgery.

[28] Yilmaz M, Vayvada H, Menderes A, Demirdover C, Barutcu A (2003). Reduction mammaplasty for phyllodes tumor causing asymmetry in an adolescent female. Breast J.

[29] Amshel CE, Sibley E (2001). Multiple unilateral fibroadenomas. Breast J.

[30] Camara O, Egbe A, Koch I, Herrmann J, Gajda M, Baltzer P, Runnebaum IB (2009). Surgical management of multiple bilateral fibroadenoma of the breast: the Ribeiro technique modified by Rezai. Anticancer Res..

[31] Maharaj D, Naraynsingh V, Ramdass M (2003). Management of giant fibroadenomas: a case for small incisions for large tumors. Breast J.

[32] Naraynsingh V, Maharaj D, Rampaul R (2002). “Swiss-roll” operation for giant fibroadenomas. Breast J.

[33] Kitamura K, Hashizume M, Sugimachi K, Kataoka A, Ohno S, Kuwano H, Maehara Y (1998). Early experience of endoscopic extirpation of benign breast tumors via an extra-mammary incision. Am J Surg.

[34] Osanai T, Ichikawa W, Takenaka S, Kojima K, Nihei Z, Sugihara K (2001). Endoscopic resection of benign breast tumors: retromammary space approach. J Jpn Soc Endosc Surg.

[35] Cheng PJ, Vu LT, Cass DL, Hicks MJ, Brandt ML, Kim ES (2012). Endoscopic specimen pouch technique for removal of giant fibroadenomas of the breast. J Pediatr Surg.

[36] Park CA, David LR, Argenta LC (2006). Breast asymmetry: presentation of a giant fibroadenoma. Breast J.

